# Urinary galectin-3 binding protein (G3BP) as a biomarker for disease activity and renal pathology characteristics in lupus nephritis

**DOI:** 10.1186/s13075-022-02763-4

**Published:** 2022-03-28

**Authors:** Huihua Ding, Yiwei Shen, Cheng Lin, Ling Qin, Shijun He, Min Dai, Shinji L. Okitsu, Julie A. DeMartino, Qiang Guo, Nan Shen

**Affiliations:** 1 Department of Rheumatology, Shanghai Institute of Rheumatology, Renji Hospital, School of Medicine, Shanghai Jiao Tong University School of Medicine, 145 Shandong (M) Rd, Shanghai, 200001 China; 2 State Key Laboratory of Drug Research, Shanghai Institute of Materia Medica, Chinese Academy of Sciences, Shanghai, China; 3 Department of Nephrology, Shanghai Tenth People’s Hospital, Tongji University School of Medicine, Shanghai, China; 4 EMD Serono (a business of Merck KGaA, Darmstadt, Germany), Billerica, MA USA; 5 China-Australia Centre for Personalized Immunology, Renji Hospital, School of Medicine, Shanghai Jiao Tong University, Shanghai, China; 6 Center for Autoimmune Genomics and Etiology, Cincinnati Children’s Hospital Medical Center, Cincinnati, OH USA; 7 Department of Pediatrics, University of Cincinnati College of Medicine, Cincinnati, OH USA

**Keywords:** Systemic lupus erythematosus, Lupus nephritis, Galectin-3 binding protein, Biomarker, Renal biopsy, Activity index

## Abstract

**Objective:**

There is an urgent need to identify novel biomarkers of LN to reflect renal histological changes. This study aims to investigate urinary G3BP levels in LN patients and their association with renal disease activity both clinically and pathologically.

**Methods:**

This is a cross-sectional study. A total of 119 lupus nephritis patients were recruited. Thirty patients with chronic kidney diseases (CKD) and 27 healthy volunteers were also recruited as controls. Urinary G3BP was tested by ELISA. Renal histopathology was reviewed by an experienced renal pathologist. Other clinical variables were collected through chart review.

**Results:**

The levels of uG3BP were significantly increased in active LN patients compared to those in inactive LN (*p*<0.001), CKD patients (*p*=0.01), and healthy controls (*p*<0.001). ROC analysis indicated a good discrimination ability of uG3BP to differentiate active LN from CKD patients (AUC=0.7), inactive LN (AUC=0.76), or healthy controls (AUC=0.87). uG3BP was positively correlated with SLEDAI (*ρ*=0.352, *p*<0.001), rSLEDAI (*ρ*=0.302, *p*<0.001), and SLICC RAS (*ρ*=0.465, *p*<0.001), indicating a role as a biomarker of disease activity. It also correlated with clinical parameters, including 24-h urine protein, ESR, and serum C3 levels. In patients with 24-h urine protein > 3.0 g/24h, uG3BP levels were higher in proliferative LN than in membranous LN (*p*=0.04). They could discriminate the two pathogenic types of LN (AUC=0.72), and they also positively correlated with AI (*ρ*=0.389, *p*=0.008) and scores of hyaline deposits (*ρ*=0.418, *p*=0.006). While in patients with 24-h urine protein ≤ 3.0 g/24h, uG3BP levels were not significantly different between proliferative and membranous LN, and there was no apparent relationship between uG3BP levels with AI or with scores of hyaline deposits, but they correlated positively with scores of cellular/fibrocellular crescents (*ρ*=0.328, *p*=0.04).

**Conclusion:**

uG3BP is a non-invasive biomarker for clinically and histologically reflecting disease activity. It is associated with active histological changes and can be used as a surrogate biomarker when the renal biopsy is impractical.

**Supplementary Information:**

The online version contains supplementary material available at 10.1186/s13075-022-02763-4.

## Introduction

Lupus nephritis (LN) is a frequent complication of systemic lupus erythematosus (SLE). LN affects approximately 60% of lupus patients, representing a major cause of mortality and morbidity in SLE [1]. Despite considerable advances in the knowledge of disease pathogenesis, standardized patient care, and improved treatment options, the rate of progression to end-stage kidney disease (ESKD) remains stable [2]. The unmet needs in the clinical management of LN include a personalized approach to assess and monitor the disease, to control the disease activity, to avoid disease flare, and to predict disease prognosis.

Prompt diagnosis and precise assessment of kidney disease are critical to the management of LN. The renal biopsy remains the gold standard for the diagnosis and management guide of LN. The value of renal biopsy in the management of LN includes establishing the histological class of glomerular disease to guide treatment and assessing the potential reversibility of active and chronic lesions to avoid over-treatment. However, it has several limitations and complications. When having a cross-sectional snapshot image of the injured kidney, the value of renal biopsy in predicting treatment response and prognosis has been challenged [3]. Due to the invasive nature, a dynamic monitoring of disease activity by serial renal biopsies is impossible and the indications of repeat biopsy in LN are controversial [4]. Traditional biomarkers, including serum creatinine and proteinuria, have been used in practice for the past few decades. They are showing a little predictive value of histological changes in LN. There is an urgent need to identify novel biomarkers that reflect histological class and activity/chronicity index in LN.

Galectin-3 binding protein (G3BP, also known as MAC-2 binding protein (M2BP) or serum protein 90K) is a secreted glycoprotein belonging to the scavenger receptor cysteine-rich (SRCR) superfamily [5]. High levels of G3BP have been detected in the serum of patients with different cancers, indicating a poorer prognosis [5–7]. In autoimmune diseases, serum G3BP has been reported to be a marker of disease activity in Behçet’s disease and SLE [8, 9]. In LN patients’ renal biopsies, G3BP co-localized with deposited immune complexes in the glomerular basement membrane, suggesting a possibility of being a biomarker in LN. However, the validation of G3BP as a biomarker in LN is lacking. The current study aimed to explore the usefulness of urinary G3BP (uG3BP) in reflecting disease activity and renal pathologic characteristics in LN patients.

## Material and methods

### Patients and study design

In this cross-sectional study, we recruited 119 lupus nephritis (LN) patients from the Department of Rheumatology at Renji Hospital, Shanghai Jiao Tong University School of Medicine, from February 2017 to August 2019. All patients fulfilled the 1997 revised American College of Rheumatology (ACR) classification criteria and had confirmed lupus nephritis. Thirty patients with chronic kidney diseases (CKD) due to causes other than lupus and proteinuria > 0.5g/24h were enrolled as disease controls. The causes of CKD include diabetic nephropathy, membranous nephropathy, IgA nephropathy, focal segmental glomerulosclerosis, and others (Table 1). The study also recruited 27 healthy volunteers as healthy controls (HC). Patients with eGFR less than 15ml/min/1.73m^2^ were excluded from the study. All study subjects are Chinese. Detailed demographic information of the study subjects is listed in Table 1.

**Table 1:** Demographic and clinical characteristics of study subjects

	Active LN	Inactive LN	CKD	HC	***P*** value^**#**^
*N*	86	33	30	27	—
Age (years), median (IQR)	34.9 (17.8)	37.0 (12.0)	62.0 (12.5)	33.0 (13.5)	0.454
Gender female, n (%)	80 (93.0%)	30 (90.9%)	12 (40.0%)	22 (81.5%)	0.696
**Disease assessment, median (IQR)**					
SLEDAI	12 (8)	4 (2)	NA	NA	<0.001
rSLEDAI	4 (4)	0 (0)	NA	NA	<0.001
SLICC RAS	11 (6)	0 (0)	NA	NA	<0.001
**System involvement,** ***n*** **(%)**					
Constitutional	15 (17.4%)	11 (33.3%)	NA	NA	0.267
Mucocutaneous	33 (38.4%)	14 (42.4%)	NA	NA	0.955
Musculoskeletal	19 (22.1%)	9 (27.3%)	NA	NA	0.679
Neuropsychiatric	0 (0.0%)	3 (9.1%)	NA	NA	0.829
Cardiorespiratory	11 (12.8%)	3 (9.1%)	NA	NA	0.460
Gastrointestinal	1 (1.2%)	4 (12.1%)	NA	NA	0.765
Renal	86 (100.0%)	33 (100.0%)	NA	NA	—
Hematological	33 (38.4%)	6 (18.2%)	NA	NA	0.175
**Laboratory tests, median (IQR)**					
ESR (mm/h)	40 (38.8)	16 (17.0)	NA	NA	<0.001
C3 (g/L)	0.6 (0.3)	0.9 (0.3)	NA	NA	<0.001
C4 (g/L)	0.1 (0.1)	0.2 (0.1)	NA	NA	0.003
eGFR (ml/min/m^2^)	97.3 (66.2)	117 (51.3)	66.3 (50.6)	NA	0.012
24-h urine protein (g/24h)	3.1 (4.0)	0.1 (0.1)	3.0 (4.9)	NA	<0.001
ANA positive/tested	75/75	10/10	NA	NA	—
Anti-U1RNP positive/tested	31/75	3/7	NA	NA	0.94
Anti-dsDNA (IU/mL)	26.1 (26.1)	21.2 (27.6)	NA	NA	0.93
**Renal disease information, n (%)**					
**LN group**					
LN II	1 (1.2%)	NA	NA	NA	—
LN III	4 (4.7%)	NA	NA	NA	—
LN III+V	11 (12.8%)	NA	NA	NA	—
LN IV	24 (27.9%)	NA	NA	NA	—
LN IV+V	21 (24.4%)	NA	NA	NA	—
LN V	23 (26.7%)	NA	NA	NA	—
Unclassified	2 (2.3%)	NA	NA	NA	—
AI, median (IQR)	5 (7)	NA	NA	NA	—
CI, median (IQR)	3 (2)	NA	NA	NA	—
**CKD group**					
DN	NA	NA	12 (40.0%)	NA	—
MN	NA	NA	5 (16.7%)	NA	—
IgAN	NA	NA	5 (16.7%)	NA	—
FSGS	NA	NA	2 (6.7%)	NA	—
Others	NA	NA	6 (20.0%)	NA	—
**Comorbidity,** ***n*** **(%)**					
Hypertension	16 (18.6%)	3 (9.1%)	NA	NA	0.147
Diabetes	1 (1.1%)	0 (0.0%)	NA	NA	0.787
Hyperlipidemia	3 (3.5%)	0 (0.0%)	NA	NA	0.638
Osteonecrosis of femoral head	8 (9.3%)	1 (3.0%)	NA	NA	0.410
Concurrent infection	7 (8.1%)	4 (12.1%)	NA	NA	0.111
**Urine G3BP/Cr (**μ**g/mg), median (IQR)**	35.5 (41.5)	14.7 (18.5)	15.1 (31.4)	7.66 (6.0)	0.003
**Urine G3BP (ng/ml), median (IQR)**	228 (267)	123 (288)	105 (222)	152 (91.9)	<0.001
**Urine Creatinine (mg/dl), median (IQR)**	71.2 (53.4)	105 (87.3)	74 (63.4)	162 (97.9)	<0.001

*LN* lupus nephritis, *CKD* chronic kidney disease, *HC* healthy control, *SLEDAI* Systemic Lupus Erythematosus Disease Activity Index, *rSLEDAI* renal SLEDAI, *SLICC RAS* SLICC renal activity score, *ESR* erythrocyte sedimentation rate, *C3* complement 3, *C4* complement 4, *eGFR* estimated glomerular filtration rate, *ANA* antinuclear antibody, *U1RNP* U1 ribonucleoprotein, *AI* activity index, *CI* chronicity index, *DN* diabetic nephropathy, *MN* membranous nephropathy, *IgAN* IgA nephropathy, *FSGS* focal segmental glomerulosclerosis

^*#*^*P* values were for comparing aLN with iLN by Mann–Whitney Wilcoxon *U* test (continuous variables) or chi-squared test/Fisher’s exact test (dichotomous variables)

All participants gave their informed consent before entering the study. The study was approved by the ethics committee of Renji Hospital, Shanghai Jiao Tong University School of Medicine, and conducted in accordance with good clinical practice.

### Disease definition and assessment

Clinical and laboratory data used in this study were retrospectively collected from patients’ admission charts at the time of sample collection. Lupus nephritis patients were further divided into active LN and inactive LN. Active LN patients were biopsy-proven and clinically had 24-h urine protein (24h UP) ≥ 0.5g/24h with or without active urine sediment. All the active LN patients had a renal SLEDAI (rSLEDAI, which refers to the total score of the urinary casts, hematuria, proteinuria, and pyuria, range 0–16) equal to or more than 4. Inactive LN patients had 24h UP < 0.5g/24h, inactive urine sediment, and stable serum creatinine. All the inactive LN patients had rSLEDAI of zero. For all the active LN patients, sample collection dates were within 7 days before the renal biopsy. Patients’ global disease activity was assessed using the Systemic Lupus Erythematosus Disease Activity Index (SLEDAI) (version SLEDAI-2k) [10]. Patients’ renal disease activity was assessed by rSLEDAI, and SLICC RAS (proteinuria 0.5–1 g/day =3 points, proteinuria >1–3 g/day =5 points, proteinuria >3 g/day=11 points; urine red blood cell count >10/hpf = 3 points; and urine white blood cell count >10/hpf =1 point; range 0–15) [11]. Renal biopsies were reviewed and classified by an experienced renal pathologist, using the revised International Society of Nephrology/Renal Pathological Society (ISN/RPS) classification [12]. For patients whose biopsies were performed before the release of the revised ISN/RPS classification, the pathologist re-assessed the slides according to the revised version. The activity index (range 0–24) and chronicity index (range 0–12) of renal pathology were calculated accordingly. Detailed clinical characteristics of the subjects are summarized in Table 1.

### Urinary G3BP measurement

The urine of the first-morning void was collected from each patient in a 50-ml sterile container. Urine samples were mixed well and aliquoted into 5-ml tubes stored at −80°C until use. In order to avoid protein degradation from multiple freeze-thaw cycles, each aliquot was retrieved and thawed only once for assays in this study. uG3BP levels were measured in urine samples by ELISA assay using human Galectin-3BP/MAC-2BP ELISA kit (DY2226) from R&D Systems (Minneapolis, MN, USA) according to the manufacturer’s instructions. All urine samples were diluted 1:30. uG3BP levels were normalized by urine creatinine levels. Urine creatinine levels were measured by Creatinine Parameter Assay Kit (KGE005, R&D Systems, Minneapolis, MN) using the same samples.

### Statistical analysis

All statistical analyses and figure plotting were performed using GraphPad Prism 7.0 (GraphPad Software Inc. La Jolla, CA, USA). Shapiro–Wilk tests established the normality of data. Continuous variables were expressed as mean (SD) for those with normal distribution or median (interquartile range (IQR)) otherwise. Dichotomous variables were expressed as counts and percentages. Comparison between the two groups was performed using Student’s *t*-test where the data were distributed normally. Otherwise, the non-parametric Mann–Whitney *U*-test was used. One-way analysis of variance (ANOVA) or Kruskal–Wallis *H* test was used to compare of three or more groups. Pearson’s or Spearman’s methods were used for correlation analysis. Linear regression was used to adjust for the patients’ demographics and clinical characteristics. Receiver operating characteristic (ROC) curve analysis was employed to evaluate the diagnostic performance of the biomarker. The area under the curve (AUC) was calculated, and the optimal cut-off point of sensitivity and specificity was determined using Youden’s index. A two-tailed value of *p* < 0.05 was considered statistically significant.

## Results

A total of 119 LN, 30 CKD, and 27 HC subjects composed the cross-sectional cohort. Among the LN patients, 86 were active LN (aLN) patients and 33 were inactive LN (iLN) patients. In the active LN patients whose urine sample collection and renal biopsy were performed concurrently, there were 1 (1.2%) class II LN, 15 (17.4%) class III/III+V LN, 45 (52.3%) class IV/IV+V, and 23 (26.7%) class V LN. Two patients’ biopsies were not classified because there were not enough glomeruli in the biopsy samples.

### Increased uG3BP in lupus nephritis patients

**Fig. 1:**
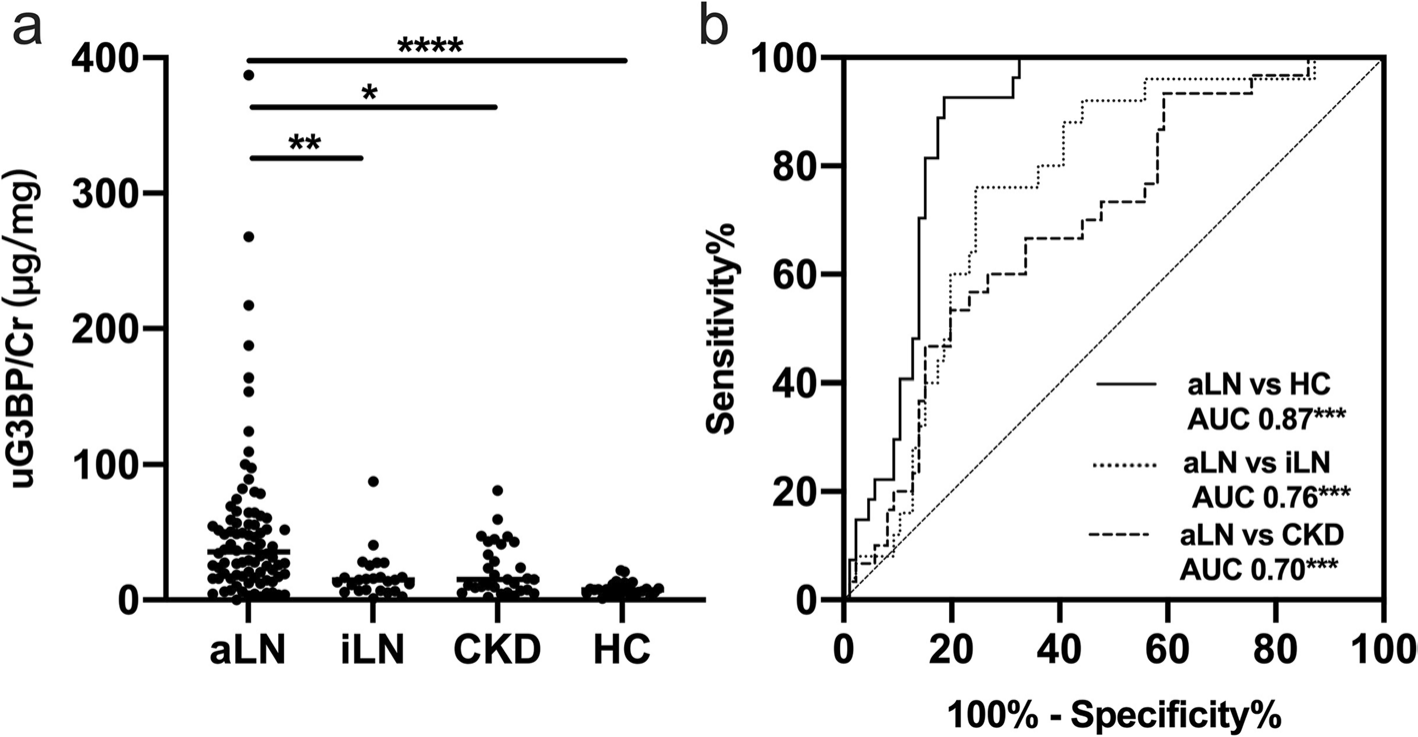
Urine G3BP levels in different groups of patients. **a** uG3BP levels were significantly increased in active LN patients (*n*=86) compared to those in inactive LN (*n*=33, *p*<0.001), CKD patients (*n*=30, *p*=0.01), and healthy controls (*n*=27, *p*<0.001). Kruskal-Wallis analysis. **b** ROC curve for uG3BP to differentiate active LN from healthy controls (AUC=0.87, *p*<0.0001, solid line), inactive LN (AUC=0.76, *p*<0.001, dotted line), and CKD patients (AUC=0.70, *p*<0.01, dashed line). aLN, active LN; iLN, inactive LN; CKD, chronic kidney disease; HC, healthy control; ROC, receiver operating characteristic; AUC, area under the curve. *p<0.05; ***p<0.001

The levels of uG3BP were significantly increased in active LN patients (35.51, IQR 17.2–58.7 μg/mg) compared to those in inactive LN (14.70, IQR 7.0–25.5 μg/mg, *p*<0.001), CKD patients (15.11, IQR 7.8–39.2 μg/mg, *p*=0.01) and healthy controls (7.66, IQR 5.1–11.1 μg/mg, *p*<0.001) (Fig. 1a). ROC analysis showed an AUC of 0.70 (95% CI 0.59–0.80) for discriminating active LN from CKD, 0.76 (95%CI 0.66–0.86) for discriminating active LN from inactive LN, and 0.87 (95% CI 0.81–0.94) for discriminating active LN from HC (Fig. 1b), indicating uG3BP could be a potential diagnostic biomarker for active LN. To identify the possible influence of some extremely high values on the results, we further analyzed the data excluding the top eight patients with the highest uG3BP levels in the active LN group. After excluding eight active LN patients with the top eight uG3BP levels, the levels of uG3BP were still significantly increased in the active LN patients (30.33, IQR 15.76–51.85 μg/mg) compared to those in inactive LN (14.70, IQR 6.83–21.22μg/mg, *p*=0.008), CKD patients (15.11, IQR 7.8–39.2 μg/mg, *p*=0.03), and healthy controls (7.66, IQR 5.1–11.1 μg/mg, *p*<0.001) (Supplemental Figure 1a). The discriminative ability of uG3BP in active LN versus inactive LN, CKD, and HC remained similar after excluding the top 8 active LN patients (Supplemental Figure 1b). The comparison of baseline characteristics between the top eight patients with the highest uG3BPlevels and the rest of the active LN patients were listed in supplemental Table 1.

### uG3BP correlated with disease activity

**Fig. 2:**
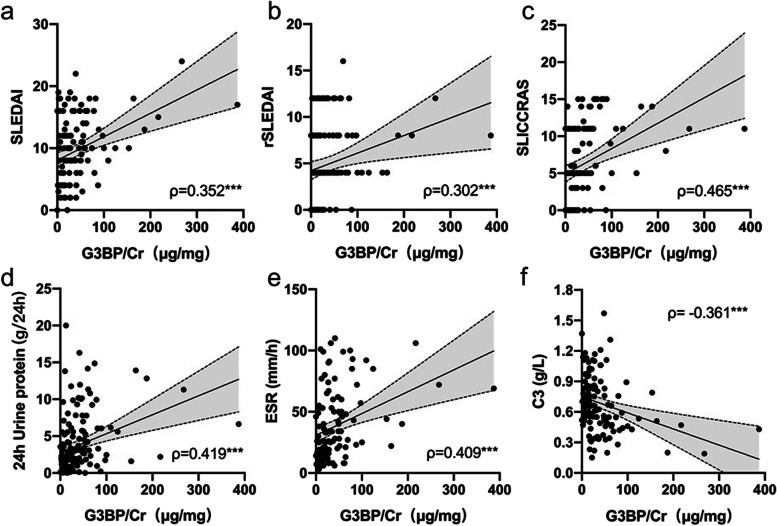
Correlation between urinary G3BP with **a** SLEDAI score, **b** rSLEDAI score, **c** SLICC RAS score, **d** 24-h urine protein, **e** ESR, and **f** complement 3. SLEDAI, Systemic Lupus Erythematosus Disease Activity Index; rSLEDAI, renal SLEDAI; SLICC RAS, SLICC renal activity score; ESR, erythrocyte sedimentation rate; C3, complement 3; ρ, Spearman’s correlation coefficient. Correlation analysis was done by Spearman’s method. ***p<0.001

As shown in Fig. 1, uG3BP levels were significantly increased in active LN patients compared to inactive LN patients. Further correlation analysis indicated a positive relationship between uG3BP and SLEDAI (*ρ*=0.352, *p*<0.001), rSLEDAI (*ρ*=0.302, *p*<0.001), and SLICC RAS (*ρ*=0.465, *p*<0.001) (Fig. 2a–c), indicating that uG3BP could be used as an indicator of general disease activity and renal disease activity in LN patients. When we looked into the individual components of the renal disease activity scores (both rSLEDAI and SLICC RAS), uG3BP positively correlated with 24-h proteinuria (*ρ*=0.419, *p*<0.001) (Fig. 2d). Correlation analysis with other lab variables showed a positive correlation of uG3BP with erythrocyte sedimentation rate (ESR) (*ρ*=0.409, *p*<0.001), and a negative correlation with complement 3 (C3) levels (*ρ*= − 0.361, *p*<0.001) (Fig. 2e, f).

### uG3BP correlated with renal pathologic characteristics

Given the potential role of uG3BP as a biomarker for disease activity in LN patients, we further explored the potential role of uG3BP in reflecting renal pathology within active LN patients. Their urine samples were collected within 7 days before the biopsy. A total of 86 active patients received renal biopsy, except for one patient with class II and two patients with unclassified LN due to insufficient glomeruli in the biopsy samples; all patients were diagnosed as class III (*n*=4), III+V (*n*=11), IV (*n*=24), IV+V (*n*=21), or V (*n*=23). Patients with class III, III+V, IV, or IV+V were considered proliferative LN patients. Class V was considered to have membranous LN.

**Fig. 3:**
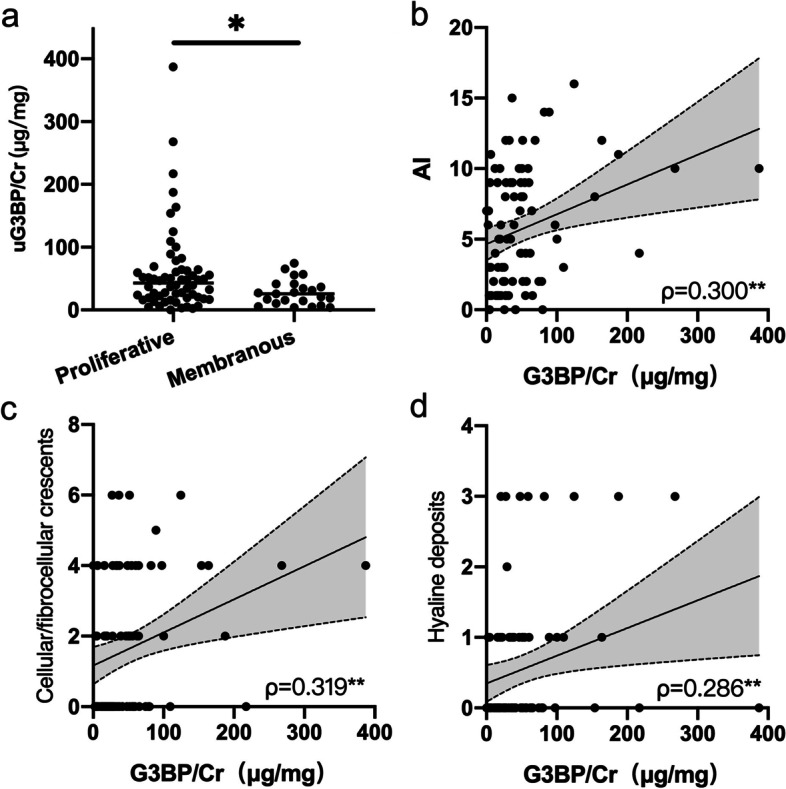
Urinary G3BP associated with renal histological changes. **a** uG3BP levels were significantly increased in proliferative (class III/IV ± V, *n*=60) LN patients, when compared to membranous LN (class V, *n*=23) (*p*=0.027) by Mann–Whitney *U* analysis. uG3BP positively correlated with concurrent (**b**) AI score (*p*=0.005) and its important components: **c** cellular/fibrocellular crescents (*p*=0.003) as well as **d** hyaline deposits (*p*=0.009). AI, activity index. *ρ*, Spearman’s correlation coefficient. Correlation analysis was done by Spearman’s method. *p<0.05; **p<0.01

When compared between proliferative and membranous LN patients, uG3BP levels were significantly higher in proliferative LN patients (43.00, IQR 19.24–63.84 μg/mg) than patients with membranous LN (25.59, IQR 10.19–41.29 μg/mg, *p*=0.027) (Fig. 3a). However, the ability of uG3BP to discriminate proliferative LN from membranous LN as measured by AUC of ROC analysis was relatively poor (AUC=0.657, *p*=0.03). uG3BP positively correlated with concurrent activity index (AI) of LN renal histology (*ρ*=0.300, *p*=0.005) (Fig. 3b), but not with chronicity index (CI) (*ρ*=−0.019, *p*=0.86). Particularly, uG3BP correlated with scores of cellular/fibrocellular crescents (*ρ*=0.319, *p*=0.003) and hyaline deposits (*ρ*=0.286, *p*=0.009), which are two important components of the AI (Fig. 3c, d).

**Fig. 4:**
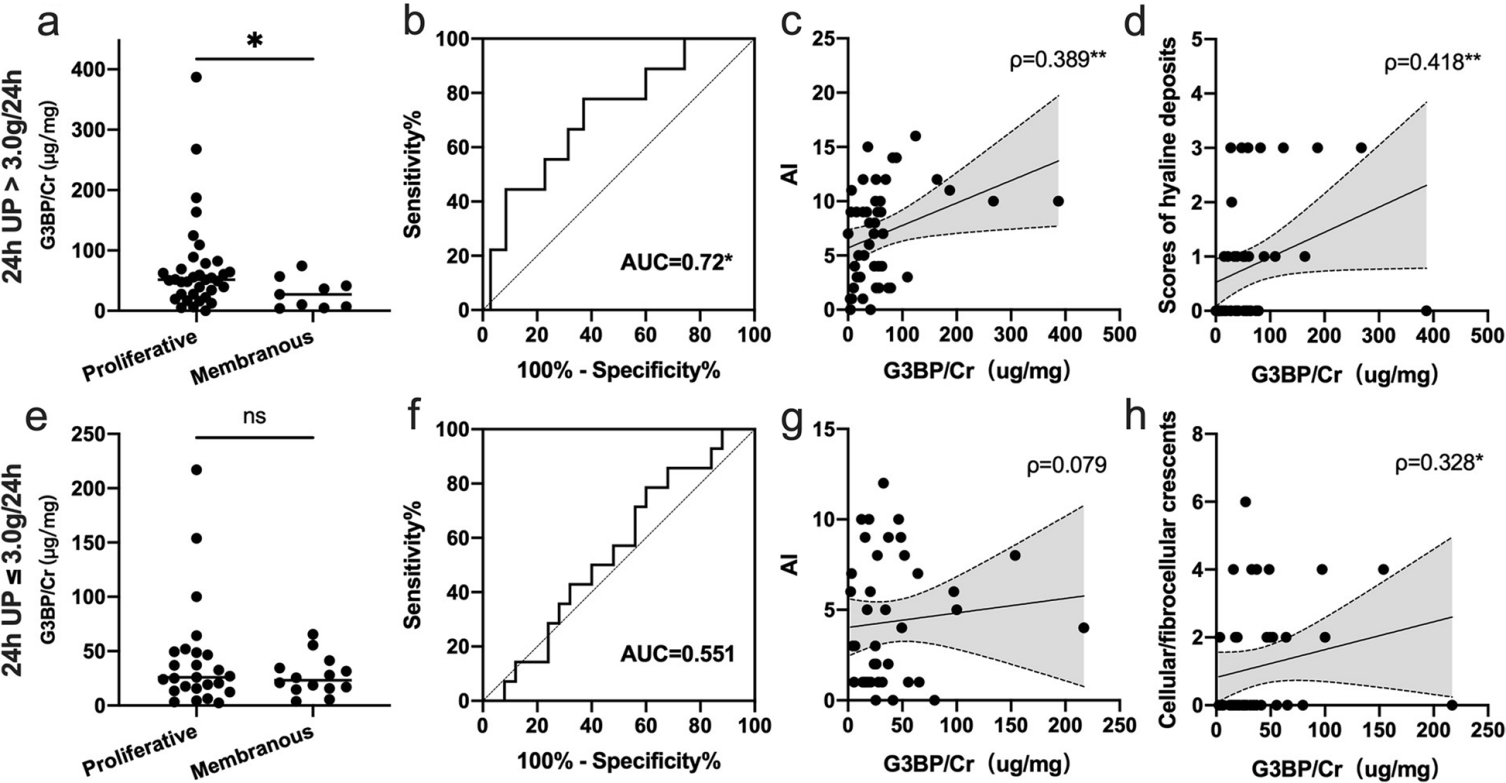
Subgroup analyses based on 24-h urine protein levels with the cut-off value of 3.0g/24h. **a**–**d** In the subgroup of patients with 24h UP>3.0 g/24h, **a** uG3BP levels were significantly increased in proliferative (class III/IV ± V, *n*=35) LN patients when compared to membranous LN (class V, *n*=9) (*p*=0.04) by Mann–Whitney *U* analysis. **b** ROC curve analysis for uG3BP to differentiate proliferative and membranous LN with AUC of 0.72 (*p*=0.04). uG3BP levels positively correlated with **c** AI score of LN renal histology (*ρ*=0.389, *p*<0.01), and **d** scores of hyaline deposits (*ρ*=0.418, *p*<0.01); **e**–**h** in the subgroup of patients with 24h UP≤3.0 g/24h, **e** there was no difference between patients with proliferative and membranous LN in uG3BP levels (*p*=0.613) by Mann–Whitney *U* analysis. **f** ROC curve analysis for uG3BP to differentiate proliferative and membranous LN with AUC of 0.551 (*p*=0.60). **g** uG3BP levels showed no correlation with AI score (*ρ*=0.079, *p*=0.627). **h** uG3BP levels correlated positively with scores of cellular/fibrocellular crescents (*ρ*=0.328, *p*<0.01). *ρ*, Spearman’s correlation coefficient. **p<0.01

To further explore the utility of uG3BP as a biomarker of renal pathology, we did subgroup analysis based on 24h UP level. In patients with 24h UP > 3.0 g/24h, uG3BP levels were significantly higher in proliferative LN (51.55, IQR 27.57–78.61 μg/mg) than in membranous patients (27.13, 5.79–49.15 μg/mg, *p*=0.04, Fig. 4a) with ROC analysis showing AUC of 0.72 (*p*=0.04, Fig. 4b). Also, in this subgroup of patients, uG3BP levels positively correlated with concurrent activity index (AI) of LN renal histology (*ρ*=0.389, *p*=0.008, Fig. 4c), and scores of hyaline deposits (*ρ*=0.418, *p*=0.006, Fig. 4d). However, in patients with 24h UP ≤ 3.0 g/24h, uG3BP levels were not significantly different between patients with proliferative and membranous LN (Fig. 4e, f). Correlation analysis showed no significant correlation between uG3BP and AI (Fig. 4g), scores of hyaline deposits. However, in patients with 24h UP ≤ 3.0 g/24h, uG3BP correlated positively with scores of cellular/fibrocellular crescents (*ρ*=0.328, *p*=0.04, Fig. 4h).

## Discussion

Renal biopsy provides critical information about the degree of active inflammation and chronic damage in the kidney. It also provides guidelines for the choice of treatment for LN. Surrogate markers, which can reflect pathological classification and activity/chronicity index, are used since serial renal biopsies in monitoring renal diseases are impossible in LN patients. The current study has validated uG3BP as a potential surrogate biomarker of disease activity and histological changes in Chinese LN patients. uG3BP levels significantly increased in active LN patients, discriminating active LN from CKD or healthy controls. uG3BP levels correlated with both general disease activity and renal disease activity. More importantly, uG3BP levels were significantly higher in proliferative LN than in membranous LN. uG3BP levels associated with AI but not CI in renal histology, particularly with AI component cellular/fibrocellular crescents and hyaline deposits. In order to assess the robustness of uG3BP as a biomarker of active LN given that 6–8 patients in the aLN group had extremely high levels of uG3BP (>100 μg/mg), we did subgroup analysis excluding the top eight patients with the highest uG3BP levels in the active LN group. The subgroup analysis showed similar results as the original data (Supplemental Figure 1). The main difference between the top eight patients and the rest of the patients in active LN patients existed in ESR levels and 24-h urine protein (UP) levels (Supplemental Table 1). Thus, subgroup analyses based on 24h UP levels were further performed for the active LN patients. The results identified that in patients with higher 24h UP (>3.0 g/24h), the uG3BP levels were higher in proliferative LN than in membranous LN, which positively correlated with AI scores and hyaline deposits in renal histology. In patients with lower 24h UP (≤ 3.0 g/24h), the uG3BP levels showed no difference in proliferative and membranous LN patients and in the correlation of AI score or hyaline deposits. Instead, they positively correlated with cellular/fibrocellular crescents. Collectively, uG3BP is a promising surrogate biomarker for monitoring disease activity and renal histological changes in LN patients.

**Fig. 5:**
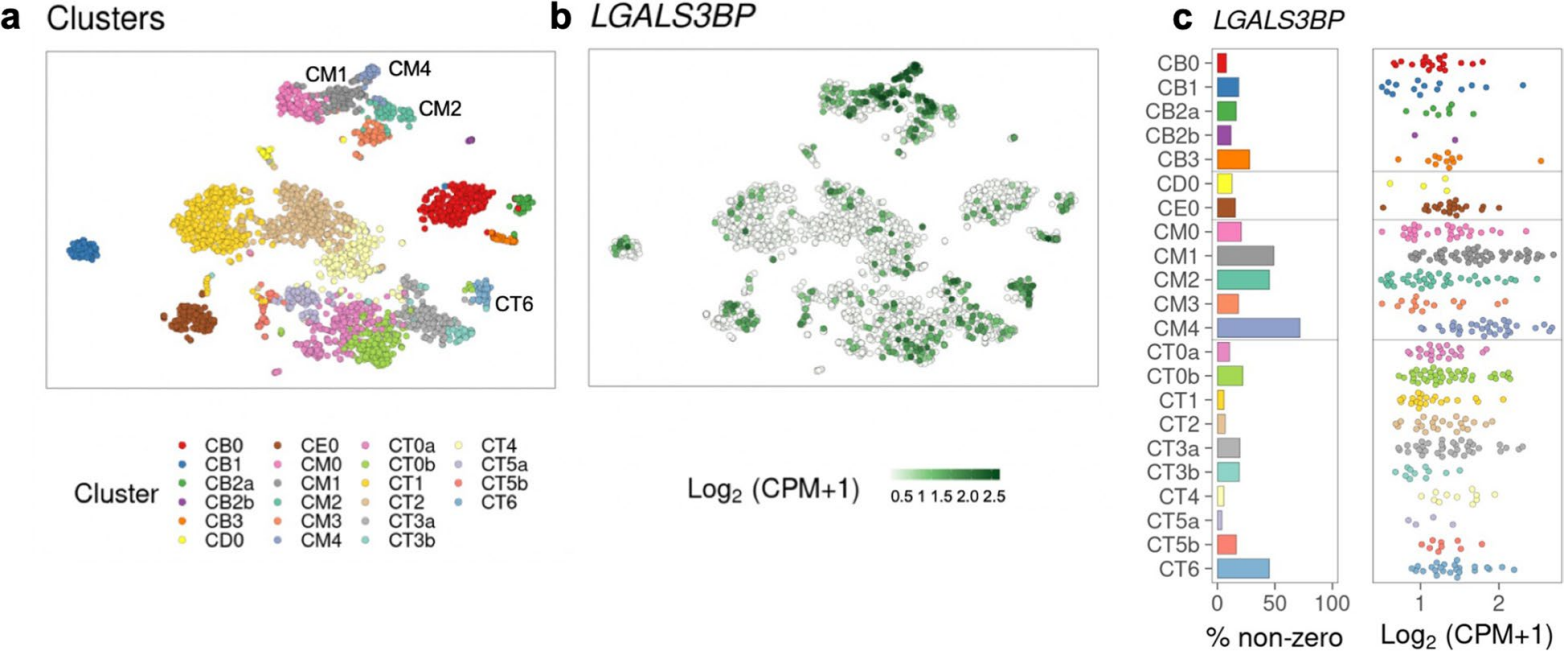
Expression spectrum of G3BP in kidneys of 24 patients with lupus nephritis. **a** Clusters of kidney cells identified with public scRNA-seq data. Cluster annotations: CM0, CD16+ machrophage, inflammatory; CM1, CD16+ macrophage, phagocytic; CM2, tissue-resident macrophage; CM3, cDCs; CM4, CD16+ macrophage, M2-like; CT0a, effector memory CD4+ T cells; CT0b, central memory CD4+ T cells; CT1, CD56_dim CD16+ NK cells; CT2, CTLs; CT3a, Tregs; CT3b, TFH-like cells; CT4, GZMK+ CD8+ T cells; CT5a, resident memory CD8+ T cells; CT5b, CD56_bright CD16- NK cells; CT6, ISG-high CD4+ T cells; CB0, activated B cells; CB1, plasma cells/plasmablasts; CB2a, naive B cells; CB2b, pDCs; CB3, ISG-high B cells; CD0, dividing cells; CE0, epithelial cells. **b**, **c** Expression of G3BP on kidney cells (https://singlecell.broadinstitute.org/single_cell/study/SCP279/amp-phase-1#study-visualize, accessed on Feb. 25, 2022)

The coding gene of G3BP is LGALS3BP, which is one of the interferon regulated genes in severe SLE patients’ PBMC [13]. Previous studies showed serum G3BP levels were significantly increased in SLE patients and associated with increased disease activity [8, 14]. G3BP was also reported as a novel circulating microparticles specific marker in SLE patients, co-localizing with deposited immune complexes in LN [15, 16]. Given the easy accessibility and complete non-invasive nature of the sample collected in urine analysis, the current study has demonstrated for the first time that G3BP levels in urine could serve as a biomarker of LN and be associated with high disease activity. We cannot determine whether the increased levels of G3BP in urine were from increased G3BP in serum or from the inflamed kidney locally since we did not collect serum samples in the study. However, public single-cell RNA-sequence data (scRNA-seq) interrogation indicated that surface G3BP is widely expressed in immune cells within LN kidneys. The top four immune cells are M2-like macrophages, phagocytic CD16+ macrophages, tissue-resident macrophages, and ISG (Interferon-stimulated gene)-high CD4+ T cells (Fig. 5) [17]. These data suggest that G3BP might be involved in the pathogenesis of lupus nephritis. Our results confirm the previously reported potential of G3BP as a biomarker of disease activity in SLE patients in a new noninvasive sample type, urine, as the biomarker source. As the ELISA for testing uG3BP is ready to be adapted to clinical laboratory setting, the translation of this study into practice is possible.

More importantly, uG3BP reflects renal biopsy results. uG3BP levels were significantly increased in proliferative (class III/III+V and IV/IV+V) LN and correlated positively with AI, particularly AI components cellular/fibrocellular crescents and hyaline deposits. Although being the gold standard of diagnosis of LN, the invasive nature of renal biopsy has limited its use in clinical practice, especially as a serial monitoring tool. High AI scores in repeat biopsies of patients with renal remission were associated with an increased chance of renal relapse in LN [18–20]. The lower percentage of cellular or fibrocellular crescents in renal biopsy was predictive of survival [21]. We envision that uG3BP will have several clinical utilities in LN patients. In patients with proteinuria higher than 3 g/24h that are reluctant or have relative contraindications to renal biopsy, a higher uG3BP level might indicate a higher probability of proliferative histological changes in the kidney, which offers guidance to the choice of treatment. And in patients with lower proteinuria (24h UP ≤ 3 g/24h), a higher uG3BP might indicate more cellular or fibrocellular crescents, showing that the patient requires more intensive treatment.

G3BP is a soluble, secreted glycoprotein that binds primarily to galectin-1 and galectin-3 [22]. It is involved in intercellular and cell-extracellular matrix adhesion and pro-inflammatory signaling [23]. Various members of cell adhesion molecules have long been reported to be increased in the circulation of SLE patients, including VCAM-1, ICAM-1, and E-selectin [24–30]. More recently, urine cell adhesion molecules such as ALCAM, VCAM-1, ICAM-1, and L-selectin have been validated as biomarkers of LN and were associated with renal histological changes [31–36]. Given the similar biological function in these molecules, the potential pathogenic role of cell-cell or cell-extracellular matrix adhesion is worth further investigation. Also, future research could consider targeting G3BP in LN treatment.

The limitations of the study include the lack of active SLE patients without renal involvement to decide whether uG3BP is specific to LN or all SLE patients with active disease. Another limitation is the lack of medication analysis in this study since no studies have ever investigated the impact of medication on the levels of uG3BP. Last but not least, the current study might have missed a group of mild active LN patients who had active sediment or a rise in creatinine but no increasement of proteinuria over 0.5 g. This is due to the use of proteinuria over 0.5 g/24h as a major definition of active LN. As indicated by Malvar et al. [37], complete clinical renal remission did not always imply histologic remission while patients with proteinuria over 0.5 g can have histological renal remission. Unfortunately, this missing group of patients rarely underwent renal biopsy at our clinical center. We believe that further validation studies with a larger sample size and better study design (i.e., prospective longitudinal study) will finally overcome these limitations.

## Conclusion

In summary, we have validated that uG3BP is a potential biomarker of LN. It specifically increased in active LN patients compared to CKD or healthy controls. It is associated with disease activity both clinically and histologically. Especially in biopsy-proven active LN patients with higher proteinuria (24h UP >3g/24h), the uG3BP could discriminate proliferative LN from membranous LN and positively correlate with AI score and hyaline deposits in renal histology.

## Supplementary Information

**Additional file 1: Supplemental Figure 1**. Urine G3BP levels in different groups of patients after excluding top 8 uG3BP level active LN patients. (a) uG3BP levels were significantly increased in active LN patients (*n*=78) compared to those in inactive LN (*n*=33, *p*=0.008), CKD patients (*n*=30, *p*=0.03) and healthy controls (*n*=27, *p*<0.001). (b) ROC curve for uG3BP to differentiate active LN from healthy controls (solid line), inactive LN (dotted line), and CKD patients (dashed line). **Supplemental Table 1**. Demographic and clinical characteristic of two subgroups of patients with active LN.

## Data Availability

The datasets generated and/or analyzed during the current study are available from the corresponding author on reasonable request.

## Abbreviations

G3BP: Galectin-3 binding protein
LN: Lupus nephritis
SLE: Systemic lupus erythematosus
CKD: Chronic kidney diseases
M2BP: MAC-2 binding protein
ACR: American College of Rheumatology
HC: Healthy controls
SLEDAI: Systemic Lupus Erythematosus Disease Activity Index
rSLEDAI: Renal SLEDAI
SLICC RAS: SLICC renal activity score
ISN/RPS: International Society of Nephrology/Renal Pathological Society
AI: Activity index
CI: Chronicity index
SD: Standard deviation
IQR: Interquartile range
ANOVA: Analysis of variance
ROC: Receiver operating characteristic
AUC: Area under the curve
aLN: Active lupus nephritis
iLN: Inactive lupus nephritis
C3: Complement 3
ACL: Anti-cardiolipin antibody
DN: Diabetic nephropathy
MN: Membranous nephropathy
IgAN: IgA nephropathy
FSGS: Focal segmental glomerulosclerosis
eGFR: Estimated glomerular filtration rate
ISG: Interferon-stimulated gene
