# Supplementary material for: Urinary galectin-3 binding protein (G3BP) as a biomarker for disease activity and renal pathology characteristics in lupus nephritis
Source: Arthritis Res Ther. 2022 Mar 28;24:77. doi: 10.1186/s13075-022-02763-4 (PMC8962213; doi:10.1186/s13075-022-02763-4)
Supplement: Supplementary file 1 — Additional file 1: Supplemental Figure 1. Urine G3BP levels in different groups of patients after excluding top 8 uG3BP level active LN patients. (a) uG3BP levels were significantly increased in active LN patients (n=78) compared to those in inactive LN (n=33, p=0.008), CKD patients (n=30, p=0.03) and healthy controls (n=27, p<0.001). (b) ROC curve for uG3BP to differentiate active LN from healthy controls (solid line), inactive LN (dotted line), and CKD patients (dashed line). Supplemental Table 1. Demographic and clinical characteristic of two subgroups of patients with active LN. [file 13075_2022_2763_MOESM1_ESM.docx]

Supplemental Table 1. Demographic and clinical characteristic of two subgroups of patients with active LN

|  | **Subgroup 1** | **Subgroup 2** | **P value^%^** |
| --- | --- | --- | --- |
| N | 8 | 78 | --- |
| Age (years), median (IQR) | 33.5 (14.3) | 34.0 (17.8) | 0.874 |
| Gender female, n (%) | 7 (87.5%) | 73 (93.6%) | 0.520 |
| BMI (mean ± SD) | 23.6 ± 4.45 | 22.0 ± 2.75 | 0.181 |
| **Disease assessment, median (IQR)** | | | |
| SLEDAI | 14.0 (7.25) | 11.0 (8.0) | 0.124 |
| rSLEDAI | 6.0 (4.0) | 4.0 (8.0) | 0.691 |
| SLICC RAS | 11.0 (1.5) | 9.0 (6.0) | 0.166 |
| **System involvement, n (%)** | | | |
| Constitutional | 3 (37.5%) | 12 (15.4%) | 0.116 |
| Mucocutaneous | 3 (37.5%) | 30 (38.5%) | 0.946 |
| Musculoskeletal | 1 (12.5%) | 18 (23.1%) | 0.691 |
| Neuropsychiatric | 0 (0%) | 2 (2.6%) | 0.647 |
| Cardiorespiratory | 2 (25%) | 9 (11.5%) | 0.513 |
| Gastrointestinal | 0 (0%) | 1 (1.3%) | 0.853 |
| Renal | 8 (100%) | 78 (100%) | --- |
| Hematological | 5 (62.5%) | 28 (35.9%) | 0.325 |
| **Laboratory tests, median (IQR)** | | | |
| ESR (mm/h) | 70.5 (43.8) | 35.0 (34.8) | **0.026** |
| C3 (g/L) | 0.45 (0.15) | 0.58 (0.36) | 0.057 |
| C4 (g/L) | 0.10 (0.046) | 0.09 (0.09) | 0.625 |
| eGFR (ml/min/m^2^) | 78.8 (56.0) | 102 (65) | 0.181 |
| 24h urine protein (g/24h) | 6.4 (6.9) | 3.02 (3.55) | **0.038** |
| ANA positive/tested | 8/8 | 67/67 | --- |
| anti-U1RNP positive/tested | 2/8 | 29/67 | 0.328 |
| anti-dsDNA (IU/mL) | 29.5 (27.5) | 23.8 (23.2) | 0.425 |
| Renal disease information, n (%) | | | |
| **LN group** |  |  | 0.370 |
| LN II | 0 (0%) | 1 (1.3%) |  |
| LN III | 0 (0%) | 4 (5.1%) |  |
| LN III+V | 2 (25%) | 9 (11.5%) |  |
| LN IV | 2 (25%) | 22 (28.2%) |  |
| LN IV+V | 4 (50%) | 17 (21.8%) |  |
| LN V | 0 (0%) | 23 (29.5%) |  |
| Unclassified | 0 (0%) | 2 (2.6%) |  |
| AI, median (IQR) | 10 (4.3) | 5 (4.1) | **0.012** |
| CI, median (IQR) | 3 (2.5) | 3 (2.1) | 0.400 |
| **Comorbidity, n (%)** |  |  |  |
| Hypertension | 2 (25%) | 7 (9.0%) | 0.158 |
| Diabetes | 0 (0%) | 1 (1.3%) | 0.747 |
| Hyperlipidemia | 0 (0%) | 3 (3.8%) | 0.572 |
| Osteonecrosis of femoral head | 1 (12.5%) | 5 (6.4%) | 0.520 |
| Concurrent infection | 2 (25%) | 5 (6.4%) | 0.067 |

^%^ P values were for comparison of subgroup 1 with 2 by Mann Whitney Wilcoxon U test (continuous variables) or Chi-Squared Test/Fisher’s exact test (dichotomous variables).


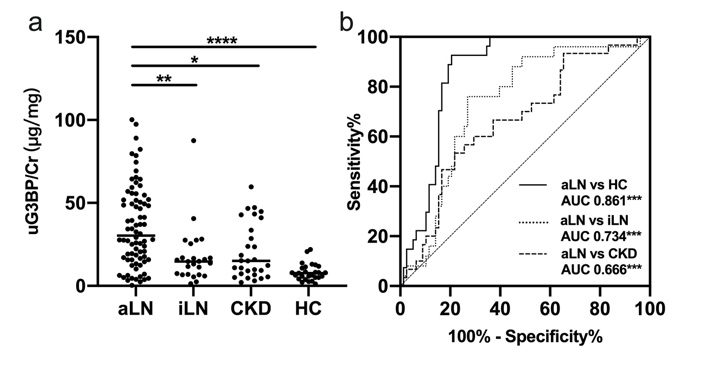


Supplemental Figure 1. Urine G3BP levels in different groups of patients after excluding top 8 uG3BP level active LN patients. (a) uG3BP levels were significantly increased in active LN patients (n=78) compared to those in inactive LN (n=33, p=0.008), CKD patients (n=30, p=0.03) and healthy controls (n=27, p<0.001). (b) ROC curve for uG3BP to differentiate active LN from healthy controls (solid line), inactive LN (dotted line), and CKD patients (dashed line).
